# Clinical relevance of Paliperidone Palmitate three-month intramuscular injection formulation: an Italian Real-World, Retrospective, one-year Mirror Image Study

**DOI:** 10.1192/j.eurpsy.2022.879

**Published:** 2022-09-01

**Authors:** S. Vanzetto, G. Cirnigliaro, V. Battini, E. Piccoli, M. Vismara, C. Viganò, B. Dell’Osso

**Affiliations:** 1 Luigi Sacco University Hospital, Psychiatry 2 Unit, ASST FBF-Sacco, University Of Milan, Milan, Italy; 2 Department of Clinical Pharmacology, ASST Fatebenefratelli-Sacco, University Of Milan, Milan, Italy; 3 “Aldo Ravelli” Center for Nanotechnology and Neurostimulation”, University Of Milan, Milan, Italy; 4 Department of Psychiatry and Behavioral Sciences, Stanford University, Milan, Italy; 5 “Centro per lo studio dei meccanismi molecolari alla base delle patologie neuro-psico-geriatriche”, University Of Milan, Milan, Italy

**Keywords:** one-year Mirror Image Study, clinical relevance, psychopharmacology, Paliperidone Palmitate 3-month formulation

## Abstract

**Introduction:**

Paliperidone Palmitate 3-month (PP3M) formulation, introduced in Italy since 2017, is an effective and safety therapeutic option for patients with schizophrenia, clinically stable with 1-month formulation (PP1M). Only a few “Real World” studies investigated the clinical relevance of PP3M and the long-term clinical and health resource utilization outcomes.

**Objectives:**

The aim of this retrospective, mirror image study was to evaluate the efficacy of PP3M in terms of continuity of care and number of hospitalizations.

**Methods:**

Fifty outpatients treated with Paliperidone Palmitate (PP) were recruited from a Community Mental Health Centre (CMHC) in Milan. Statistical analysis were conducted with SPSS 26. Frequencies of hospitalization 6 months before and after the start of PP3M were compared using the McNemar test, setting the significance to p<0.05.

**Results:**

This study involved 34 patients (68%) treated with PP1M and 16 (32%) treated with PP3M.The median time interval between PP1M and PP3M was 14 months. After the switch to PP3M, 69% of patients continued to visit the CMHC with an unchanged frequency (50% once/month, 6% more than once/month), while 31% with a decreased frequency (once/3 months). No patient increased the frequency of CMHC visits or started visiting it discontinuously. 44% of subjects had had at least one hospitalization prior to the switch and no hospitalizations after (p=0.016). Moreover, no patients showed increased hospitalizations

**Conclusions:**

In this study PP3M clinical relevance was confirmed comparing pre-initiation and post-initiation 6-months time intervals: hospitalizations number significantly decreased, while the continuity of care was preserved. Further studies on a greater sample are necessary to support these preliminary data.

**Disclosure:**

No significant relationships.

